# BMS-599626, a Highly Selective Pan-HER Kinase Inhibitor, Antagonizes ABCG2-Mediated Drug Resistance

**DOI:** 10.3390/cancers12092502

**Published:** 2020-09-03

**Authors:** Yunali V. Ashar, Jingchun Zhou, Pranav Gupta, Qiu-Xu Teng, Zi-Ning Lei, Sandra E. Reznik, Sabrina Lusvarghi, John Wurpel, Suresh V. Ambudkar, Zhe-Sheng Chen

**Affiliations:** 1 Department of Pharmaceutical Sciences, College of Pharmacy and Health Sciences, St. John’s University, Queens, NY 11439, USA; yunali.ashar17@stjohns.edu (Y.V.A.); Pra.1391@gmail.com (P.G.); qiuxu.teng15@stjohns.edu (Q.-X.T.); zining.lei14@stjohns.edu (Z.-N.L.); rezniks@stjohns.edu (S.E.R.); wurpelj@stjohns.edu (J.W.); 2 Department of Otorhinolaryngology, Shenzhen People’s Hospital (The Second Medical College, Jinan University; The First Affiliated Hospital, Southern University of Science and Technology), Shenzhen 518020, Guangdong, China; zhou.jingchun@szhospital.com; 3 Departments of Pathology and Obstetrics and Gynecology and Women’s Health, Albert Einstein College of Medicine, Bronx, NY 10461, USA; 4 Laboratory of Cell Biology, Center for Cancer Research, National Cancer Institute, NIH, Bethesda, MD 20892, USA; sabrinal@mail.nih.gov (S.L.); ambudkar@mail.nih.gov (S.V.A.)

**Keywords:** BMS-599626, ABC transporters, ABCG2, multidrug resistance, chemotherapy, HER kinase inhibitor

## Abstract

**Simple Summary:**

ABC transporters comprise a large group of ATP binding plasma membrane proteins, classified into subfamilies A-G, that transport substrates out of cells to maintain homeostasis. Prolonged exposure to chemotherapeutic drugs leads to increased expression of ABC transporters in cancer cells, resulting in increased efflux and decreased efficacy of anti-neoplastic agents. We found that BMS-599626, at 300 nM, inhibited the function of ABCG2, thereby increasing the efficacy of substrate chemotherapeutic drugs in wild-type as well as mutant ABCG2 overexpressing cells. In addition, BMS-599626 did not alter the expression or intracellular localization of ABCG2 but produced its reversal effect by decreasing efflux and increasing the intracellular accumulation of substrate chemotherapeutic drugs. Finally, BMS-5999626 also inhibited ABCG2 mediated ATP hydrolysis. Overall, our results show that administration of BMS-599626 along with chemotherapeutic drugs can improve the efficacy of chemotherapy in ABC transporter overexpressing cancer cells.

**Abstract:**

Multidrug resistance (MDR) associated with the overexpression of ABC transporters is one of the key causes of chemotherapy failure. Various compounds blocking the function and/or downregulating the expression of these transporters have been developed over the last few decades. However, their potency and toxicity have always been a concern. In this report, we found that BMS-599626 is a highly potent inhibitor of the ABCG2 transporter, inhibiting its efflux function at 300 nM. Our study repositioned BMS-599626, a highly selective pan-HER kinase inhibitor, as a chemosensitizer in ABCG2-overexpressing cell lines. As shown by the cytotoxicity assay results, BMS-599626, at noncytotoxic concentrations, sensitizes ABCG2-overexpressing cells to topotecan and mitoxantrone, two well-known substrates of ABCG2. The results of our radioactive drug accumulation experiment show that the ABCG2-overexpressing cells, treated with BMS-599626, had an increase in the accumulation of substrate chemotherapeutic drugs, as compared to their parental subline cells. Moreover, BMS-599626 did not change the protein expression or cell surface localization of ABCG2 and inhibited its ATPase activity. Our in-silico docking study also supports the interaction of BMS-599626 with the substrate-binding site of ABCG2. Taken together, these results suggest that administration of chemotherapeutic drugs, along with nanomolar concentrations (300 nM) of BMS-599626, may be effective against ABCG2-mediated MDR in clinical settings.

## 1. Introduction

For the last several decades, chemotherapeutic agents have remained the most successful drugs for the treatment of solid and blood malignancies [1,2,3,4,5]. However, chronic use of these agents leads to a decreased sensitivity of cancer cells to these drugs; this is referred to as multidrug resistance (MDR) [2,3,6]. Thus, continuous treatment with chemotherapeutic drugs leads to the development of certain mechanisms in cancer cells, making them less sensitive to treatment with the same drugs [2,7,8,9]. MDR is the principal factor leading to the failure of chemotherapy and therefore, overcoming MDR remains a major obstacle to effective treatment with chemotherapeutic drugs [4,6,10].

Several extrinsic and intrinsic mechanisms causing MDR have been identified [6,11,12,13,14]. Amongst these, the increased cell surface expression of ATP-binding cassette transporters (ABC transporters) causing efflux of chemotherapeutic agents is a major cause [1,2,3,6,13,15,16,17]. There are 49 ABC transporters, divided into subfamilies from A to G [2]. ABCG2, also known as breast cancer resistance protein (BCRP) and mitoxantrone resistance (MXR) transporter, is one of these 49 transporters, and remains one of the most extensively studied transporters [18,19]. It is a 72 kDa protein with the highest concentrations in the brain, kidneys, intestines, placenta, and colon. Its function is to efflux harmful and toxic chemicals out of cells in these vital organs [2,3,18,20]. Long-term treatment with chemotherapeutic agents may lead to an increase in the expression of ABCG2 in cancer cells, causing an increase in the efflux and decrease in the intracellular accumulation of its substrates, thereby causing MDR [21,22,23,24].

In the past four decades, various drugs inhibiting the function of ABC transporters have been developed, thereby circumventing ABC transporter-mediated MDR [23]. BMS-599626 is a very potent pan-HER kinase inhibitor [13]. It is a pyrrolotriazine-based compound which inhibits HER-1 and HER-2 with an IC_50_ of 20 to 30 nM [25]. It also inhibits HER-3 to a lesser extent. Heterodimerization of HER1/HER2 is inhibited by BMS-599626, which further blocks downstream signaling [25]. However, the ability of BMS-599626 to overcome ABC transporter-mediated MDR and chemosensitize ABCG2-overexpressing cells has not been explored. Here, we report that BMS599626 inhibits the efflux activity of ABCG2 at nontoxic, low nanomolar concentrations, thereby increasing the chemosensitivity of cancer cells to their substrate chemotherapeutic agents.

## 2. Results

### 2.1. Cytotoxicity Study of BMS-599626 in Parental and ABCB1-, ABCG2-, ABCC1-, and ABCC10-Overexpressing Cells

**Figure 1:**
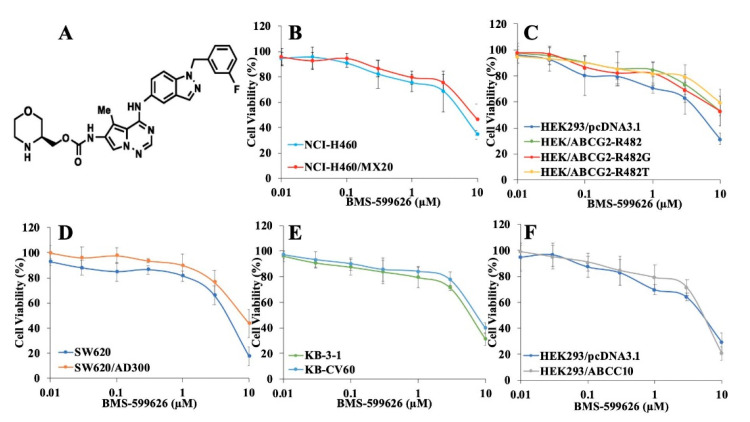
Structure and cytotoxicity curves of BMS-599626 in parental and ABCB1-, ABCG2-, ABCC1-, and ABCC10-overexpressing cells. (**A**) Two-dimensional structure of BMS-599626: [4-[[1-(3-fluorophenyl)methyl]-1H-indazol5-ylamino]-5-methylpyrrolo [2,1-f][1,2,4]triazin6-yl]carbamic acid, (3S)-3-morpholinylmethyl ester, (**B**) cell viability vs. concentration curve of NCI-H460 and resistant NCI-H460/MX20 cells, (**C**) cell viability vs. concentration curve of HEK293/pcDNA3.1 and wild type ABCG2-expressing HEK/ABCG2-R482, and its mutants HEK/ABCG2-R482G, and HEK/ABCG2-R482T cells, (**D**) cell viability vs. concentration curve of SW620 and resistant SW620/AD300 cells, (**E**) cell viability vs. concentration curve of KB-3-1 and resistant KB-CV-60 cells and (**F**) cell viability vs. concentration curve of HEK293/pcDNA3.1 and resistant HEK293/ABCC10 cells. Mean values from three independent experiments are given and error bars indicate SD.

Before studying BMS-599626 (chemical structure in Figure 1A) for its chemosensitizing function, we evaluated the cytotoxicity of this compound using an MTT assay in parental and ABCG2-overexpressing wild-type and mutant cell lines. In addition, cytotoxicity studies were also performed on SW620 and ABCB1-overexpressing SW620/AD300 cell lines, KB-3-1 and ABCC1-overexpressing KB-CV60 cell lines, and HEK293/pcDNA3.1 and ABCC10-overexpressing HEK293/ABCC10 cell lines. As shown in Figure 1B–F, BMS-599626 is non-cytotoxic (cell survival more than 80% in all cell lines) at 100 and 300 nM. These concentrations were used for further studies.

### 2.2. BMS-599626 Increases the Sensitivity of ABCG2-Overexpressing Cells to the Substrates of ABCG2

**Figure 2:**
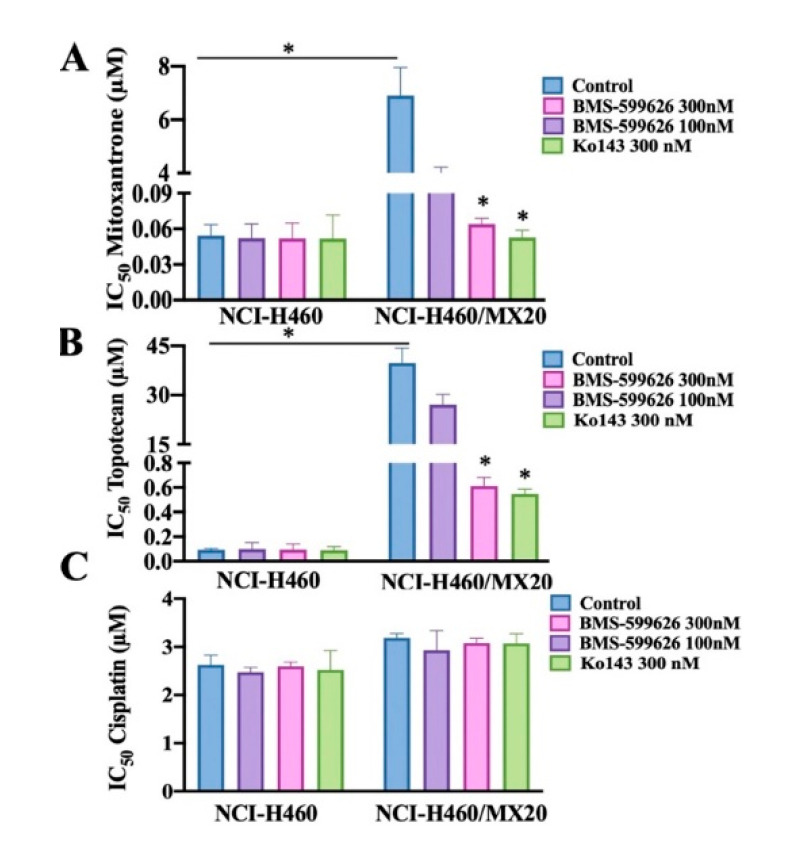
Effect of BMS-599626 on multidrug resistance (MDR) in parental and ABCG2-overexpressing cells. (**A**) The IC_50_ of mitoxantrone in NCI-H460 and NCI-H460/MX20 cells with or without BMS-599626 and Ko143, (**B**) the IC_50_ of topotecan in NCI-H460 and NCI-H460/MX20 cells with or without BMS-599626 and Ko143, (**C**) the IC_50_ of cisplatin in NCI-H460 and NCI-H460/MX20 cells with or without BMS-599626 and Ko143. Mean values from three independent experiments are given and error bars indicate SD. (*) indicates *p* < 0.05 compared to control group.

The chemosensitizing activity of BMS-599626 was evaluated by the MTT cell survival assay. Mitoxantrone and topotecan were used as ABCG2 substrates, and since cisplatin is not a substrate of ABCG2, it was considered a negative control anticancer agent. As shown in Figure 2, the IC_50_ values of mitoxantrone and topotecan in ABCG2-overexpressing cells were higher than those of the parental cells. Upon treatment with 100 and 300 nM BMS-599626, there was a significant reduction in the IC_50_ values of substrate drugs in ABCG2-overexpressing cells, but the IC_50_ remained unchanged in the parental NCI-H460 cells. These findings are comparable to Ko143, which is a known inhibitor of ABCG2 [26,27]. There was no change in the IC_50_ values of cisplatin upon treatment with 100 and 300 nM BMS-599626 or 300 nM Ko143.

### 2.3. BMS-599626 Increases the Sensitivity of ABCG2-Overexpressing Transfected Cells to the Substrates of ABCG2

**Figure 3:**
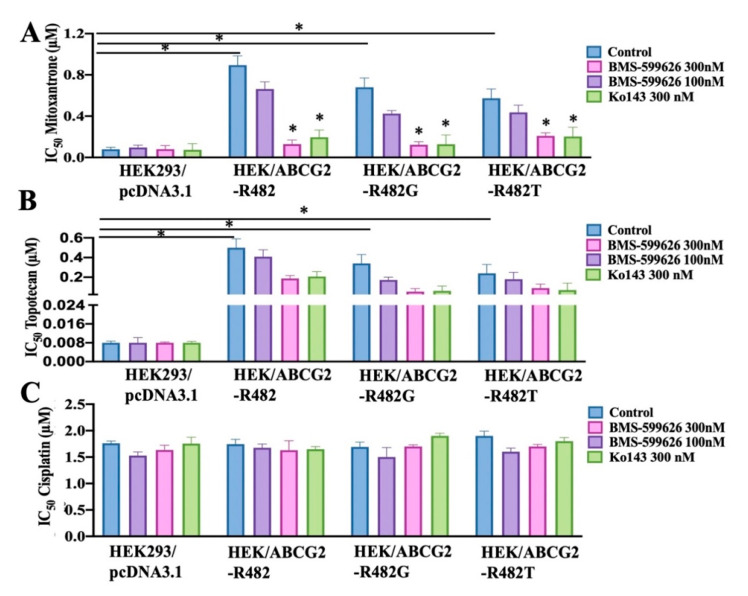
Effect of BMS-599626 on MDR in transfected cell lines overexpressing mutant ABCG2. (**A**) The IC_50_ of mitoxantrone in HEK293/pcDNA3.1 and wild-type HEK/ABCG2-R482, mutant HEK/ABCG2-R482G, and HEK/ABCG2-R482T cells with or without BMS-599626 or Ko143, (**B**) the IC_50_ of topotecan in HEK293/pcDNA3.1 and wild-type HEK/ABCG2-R482, mutants HEK/ABCG2-R482G, and HEK/ABCG2-R482T cells with or without BMS-599626 or Ko143, (**C**) the IC_50_ of cisplatin in HEK293/pcDNA3.1 and wild-type HEK/ABCG2-R482, mutant HEK/ABCG2-R482G, and HEK/ABCG2-R482T cells with or without BMS-599626 or Ko143. Mean values from three independent experiments are given and error bars indicate SD. (*) indicates *p* < 0.05 compared to control group.

Polymorphisms and mutations of ABCG2 might change the mechanism and substrate recognition of this transporter [28,29,30]. This can lead to alterations in substrate specificity and transporter activity. Mutations in R482 in the ABCG2 primary sequence significantly modify substrate specificity [28,31,32]. To study the specificity of BMS-599626, its ability to block the efflux function of ABCG2 in R482 and its variants, R482G and R482T, was evaluated. As shown in Figure 3, there was a significant decrease in the IC_50_ values of substrate drugs upon treatment with BMS-599626 in wild-type HEK/ABCG2-R482 and variants HEK/ABCG2-R482G and HEK/ABCG2-R482T cells, suggesting decreased drug resistance and increased sensitivity of these cells to ABCG2 substrates.

### 2.4. BMS-599626 Does Not Sensitize ABCB1- or ABCC1-Overexpressing Cells to Their Respective Chemotherapeutic Agents, but Partially Sensitizes ABCC10-Overexpressing Cells

To further evaluate the selectivity of BMS-599626 to ABCG2, we studied its inhibitory activity on cells overexpressing ABCB1, ABCC1, and ABCC10 transporters. Doxorubicin was used as a substrate of ABCB1 and vincristine was used as a substrate of ABCC1 and ABCC10. Verapamil, MK571, and cepharanthine were used as positive controls for ABCB1, ABCC1, and ABCC10, respectively. As shown in Table 1, there was no significant reduction in the IC_50_ values of doxorubicin in ABCB1-overexpressing cells or vincristine in ABCC1-overexpressing cells upon treatment with BMS-599626, at 100 and 300 nM. On the other hand, BMS-599626 produced a partial decrease in the IC_50_ of vincristine in cells overexpressing ABCC10, suggesting that BMS-599626 partially sensitizes ABCC10-overexpressing cells to their substrate.

### 2.5. BMS-599626 Increases the Intracellular Accumulation of [^3^H]-Mitoxantrone in ABCG2-Overexpressing Cell Sublines

**Figure 4:**
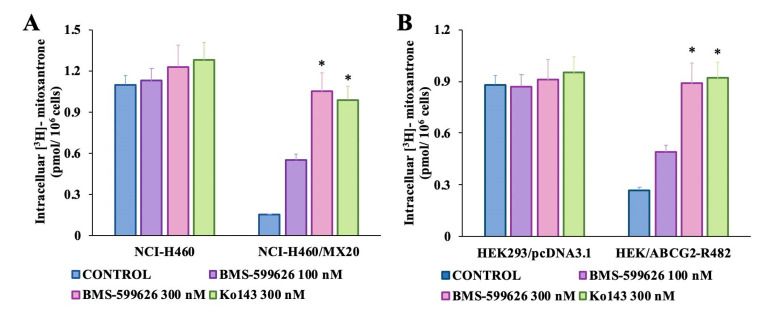
Effect of BMS-599626 on the intracellular accumulation of [^3^H]-mitoxantrone in parental cells and cells overexpressing wild type and mutant ABCG2. Effect of BMS-599626 on the intracellular accumulation of [^3^H]-mitoxantrone in (**A**) NCI-H460 and NCI-H460/MX20 cells and (**B**) HEK293/pcDNA3.1 and HEK/ABCG2-R482 cells. Mean values from three independent experiments are given and error bars indicate SD. (*) indicates *p* < 0.05 compared to the control group.

The effect of treatment with 100 and 300 nM BMS-599626 on the accumulation of [^3^H]-mitoxantrone in parental and ABCG2-overexpressing cells was evaluated. As shown in Figure 4A,B, there was a concentration-dependent increase in the intracellular accumulation of [^3^H]-mitoxantrone upon treatment with 100 and 300 nM BMS-599626 in ABCG2-overexpressing cells as compared to control. The increase in the accumulation of [^3^H]-mitoxantrone was higher upon treatment with 300 nM BMS-599626 compared with Ko143 in the NCI-H460/MX20-resistant cell line. In addition, in HEK/ABCG2-R482 cells, the accumulation of [^3^H]-mitoxantrone upon treatment with 300 nM BMS-599626 was comparable to that of Ko143.

### 2.6. BMS-599626 Decreases the Efflux of [^3^H]-Mitoxantrone in ABCG2-Overexpressing Cells

**Figure 5:**
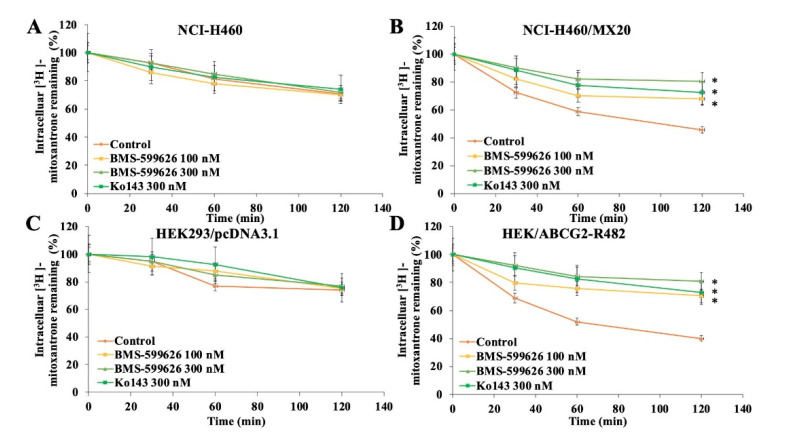
Effect of BMS-599626 on the efflux of [^3^H]-mitoxantrone. The time course (0, 30, 60 and 120 min) vs. percent of intracellular [^3^H]-mitoxantrone in (**A**) NCI-H460 cells, (**B**) NCI-H460/MX20 cells, (**C**) HEK293/pcDNA3.1 cells, and (**D**) HEK/ABCG2-R482 cells. Ko143 was used as a positive control. Mean values from three independent experiments are given and error bars indicate SD. (*) indicates *p* < 0.05 compared to control group.

To determine whether the increase in the intracellular accumulation of [^3^H]-mitoxantrone was due to a decrease in the efflux function of ABCG2, the efflux activity of ABCG2 was evaluated in the presence of BMS-599626. Figure 5 shows that prior to treatment with BMS-599626, ABCG2-overexpressing cell lines showed a significantly higher efflux activity compared to parental cell lines. Upon treatment with 100 and 300 nM BMS-599626, there was a greater inhibition of the efflux of [^3^H]-mitoxantrone at 300 nM, compared with Ko143 in ABCG2-overexpressing cell lines.

### 2.7. BMS-599626 Does not Change the Total or Cell Membrane Expression of ABCG2

**Figure 6:**
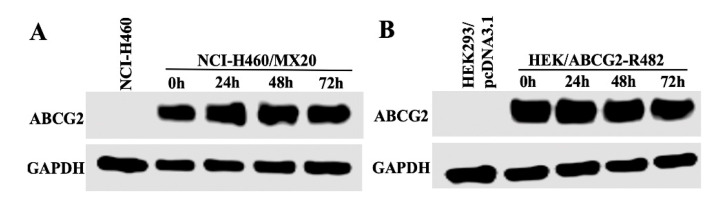

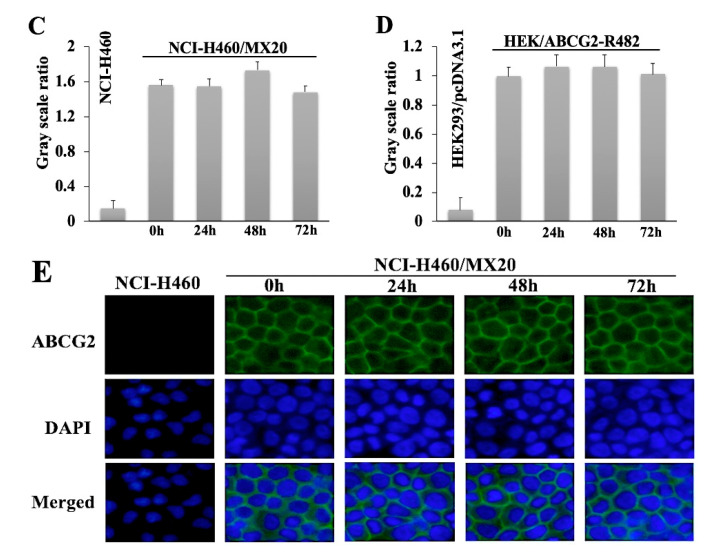
Effect of BMS-599626 on the protein expression and subcellular localization of ABCG2. Effect on the expression of ABCG2 protein after treatment with 300 nM BMS-599626 at different time points (0, 24, 48 and 72 h) in (**A**) NCI-H460/MX20 and (**B**) HEK/ABCG2-R482 cells. GAPDH was used as a loading control and equal amounts of protein were loaded for each sample. (**C**,**D**) Quantification of the protein expression was done by gray scale values. Error bars are representative of mean ± SD from 3 independent experiments. (**E**) ABCG2 protein expression in NCI-H460 and NCI-H460/MX20 cells after incubation with 300 nM BMS-599626 for different time periods (0, 24, 48 and 72 h). DAPI was used to counterstain the nuclei. Images have been modified by Photoshop software for merged comparison.

Expression levels of ABCG2 upon treatment with BMS-599626 were determined. Our results indicate that the expression level of ABCG2 in the resistant NCI-H460/MX20 (Figure 6A,C) and HEK/ABCG2-R482 (Figure 6B,D) cells remained unchanged upon treatment with 300 nM BMS-599626 for 72 h. Whole uncut blots are shown in Figure S1.

We further evaluated whether BMS-599626 changed the expression and cellular localization of ABCG2 using the immunofluorescence assay. The cells were treated with 300 nM BMS-599626 at different time points. As demonstrated in Figure 6E, the ABCG2 transporters are localized on the cell membrane of the resistant NCI-H460/MX20 cells. On incubating the cells with 300 nM BMS-599626, there was no significant alteration in the subcellular distribution of ABCG2 when examined at different time points (0, 24, 48 and 72 h). The effect of long-term treatment of BMS-599626 on protein expression or sub-cellular localization warrants further investigation.

### 2.8. BMS-599626 Inhibits the ATPase of ABCG2

**Figure 7:**
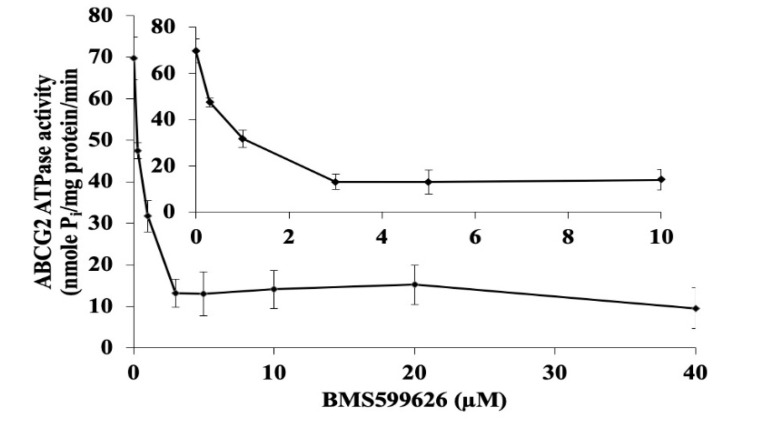
Effect of BMS-599626 on the ATPase activity of ABCG2. The inset shows inhibition of ATP hydrolysis at concentrations of 0–10 µM BMS-599626. The mean values are plotted, and error bars depict SD obtained from the average of at least three independent experiments.

To ascertain whether BMS-599626 inhibits the ATPase activity of ABCG2, we evaluated the effect of BMS-599626 on ABCG2-mediated ATP hydrolysis. Our results show that BMS-599626 inhibits the vanadate-sensitive ATPase activity of ABCG2 (Figure 7) in a concentration-dependent manner with 7.6-fold inhibition of basal activity.

### 2.9. Molecular Docking Analysis of the Interaction between BMS-599626 and ABCG2: BMS-599626 Inhibits the ATPase Activity of ABCG2

**Figure 8:**
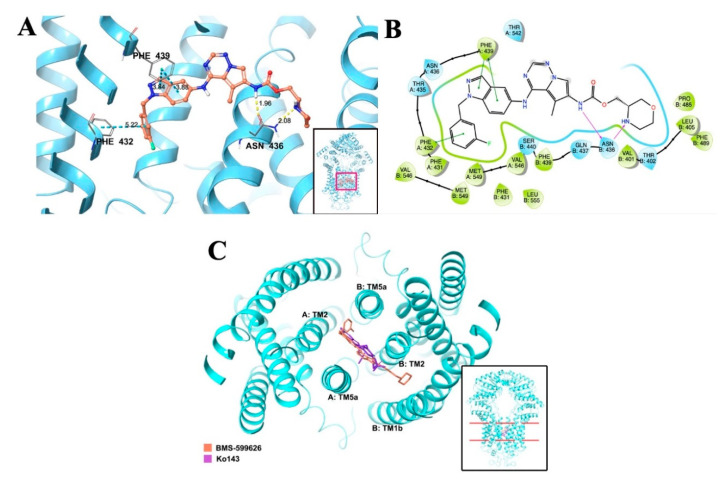
Induced-fit docking (IFD) predicted the best-scored binding pose of BMS-599626 in human ABCG2 model. (**A**) Overview of the binding location of BMS-599626 (with crimson surface) in human ABCG2 (cyan ribbon diagram) and the detailed bonding interactions are shown. The location of BMS-599626 within the drug-binding cavity shown as a ball and stick model with the following colors: carbon, orange; hydrogen, white; oxygen, red; nitrogen, blue; and fluorine, green. Important amino acids are depicted as sticks in the same scheme except with carbon as grey. Dotted yellow lines and dotted blue lines are indicative of hydrogen-bonding interactions and π-π stacking interactions, respectively. Values of the relevant distances are provided in Å. (**B**) The two-dimensional ligand-receptor interaction diagram of BMS-599626 and human ABCG2. The amino acids within 4 Å are shown as colored bubbles, polar residues are shown in blue color, and hydrophobic residues are indicated in green color. Hydrogen bonds and π-π stacking aromatic interactions are shown by the purple arrow and green lines, respectively. (**C**) The docked conformations of BMS-599626 (orange) and Ko143 molecules (purple) into human ABCG2 (6ETI) as stick model is magnified for better view. Important interacting TMs of ABCG2 are labeled.

The best docking pose of BMS-599626 with human ABCG2 (induced-fit docking (IFD) Glide gscore: −12.886 kcal/mol) is shown in Figure 8A,B. The predicted ABCG2–BMS-599626 complex showed that the position of BMS-599626 was most stable at the transmembrane domain of ABCG2 [31]. Hydrogen-bonding interactions mostly occurred between Asn436 in chain B of ABCG2 and the nitrogen atoms in the morpholine ring, as well as in the carbamoyl group of BMS-599626. In addition, π-π stacking interactions with the nearby aromatic residues Phe432 and Phe439 on chain A of ABCG2 were predicted, involving the fluorophenyl ring and the indazole group in BMS-599626, respectively. BMS-599626 was predicted to be stabilized in the drug-binding site of human ABCG2 by hydrophobic interactions with residues Phe431, Phe432, Phe439, Val546, and Met549 at chain A, and Val401, Leu405, Phe431, Phe439, Pro485, Phe489, Val546, and Met549 at chain B. Moreover, our results show that the predicted binding site of BMS-599626 is identical to that of Ko143 at the transmembrane (TM) ligand binding cavity, with similar hydrophobic interactions with ABCG2 TM helixes TM1b of chain B as well as TM2 and TM5 of both chains (Figure 8C). This indicates that BMS-599626, similar to Ko143, can inhibit ABCG2 by blocking access for substrates and hindering conformational changes, thereby reducing ATPase activity.

## 3. Discussion

One of the major obstacles in cancer pharmacology is that, with chronic exposure, some tumors develop resistance to chemotherapeutic agents. This drug resistance cannot be reversed even with administration of higher doses of these drugs. There is much evidence that overexpression of ABC transporters leads to MDR [2,4,6,33,34], and that this phenomenon results in increased morbidity and mortality in cancer patients [14,22,24]. This drug resistance can be circumvented by using chemotherapeutic agents that are not substrates of ABC transporters, which unfortunately most of the clinically available first-line chemotherapeutic drugs are. Therefore, using small molecules called “chemosensitizers”, which inhibit ABC transporters, along with the substrate drugs, may be a potential treatment strategy which could bypass MDR [33]. Over the last several decades, various compounds that increase the efficacy of chemotherapeutic agents by inhibiting the activity of ABC transporters have been reported [11,18,35].

BMS-599626 is highly selective and a very potent pan-HER kinase inhibitor, inhibiting HER1 with an IC_50_ of 20 nM and HER2 with an IC_50_ of 30 nM [25]. However, its potential to reverse ABCG2-mediated MDR has not been reported. The majority of the compounds developed and tested for reversing ABC transporters exhibit their activity in the micromolar range [8,10,18]. One of the key and novel findings of this study is the ability of BMS-599626, at concentrations as low as 100 and 300 nM, to sensitize wild-type and mutant cells overexpressing ABCG2 to substrate drugs. We determined the non-cytotoxic concentrations of BMS-599626 to be 100 and 300 nM, which were then used for our reversal experiments. As evident from our reversal studies, treatment with BMS-599626 at 100 and 300 nM significantly reduced the IC_50_ values of mitoxantrone and topotecan in NCI-H460/MX20, which are ABCG2-overexpressing cells. The positive control inhibitor, Ko143, showed comparable inhibition. Studies in the past have shown such comparable inhibition of ABCG2 drug efflux activity [11,36]. Although BMS-599626 sensitized the NCI-H460/MX20 cell line to substrate chemotherapeutic drugs, it did not significantly sensitize SW620/AD300 cells (cells overexpressing ABCB1) or KB-CV60 cells (cells overexpressing ABCC1) to doxorubicin and vincristine, respectively. Moreover, BMS-599626 partially increased the sensitivity of HEK293/ABCC10 cells to vincristine, suggesting a partial inhibitory effect on ABCC10. These results indicate that BMS-599626 specifically inhibits ABCG2.

Position 482 is important for the function of the ABCG2 transporter to identify and recognize drug substrates [29,37]. Polymorphisms in this position can alter the drug specificity, drug and substrate recognition, and transport function of ABCG2 [37]. While overcoming ABCG2-mediated MDR in wild-type ABCG2-overexpressing cells is achievable, inhibition of mutant forms of ABCG2 is a challenge. Recently, the compound venetoclax blocked the function of only wild-type ABCG2 and not the mutant ABCG2 [8]. This shows that mutations in ABCG2 might result in a change in the transporter function. Therefore, it was important to examine whether BMS-599626 sensitized the mutant cell lines to the substrates of ABCG2. Our results demonstrate that BMS-599626 inhibits wild-type ABCG2 as well as its mutant cell lines HEK/ABCG2-R482G and HEK/ABCG2-R482T, as shown by an increase in the sensitivity of cell lines to mitoxantrone and topotecan. In addition, BMS-599626 did not sensitize the parental or ABCG2-overexpressing cell lines to cisplatin.

We further determined the mechanism by which BMS-599626 potently inhibits ABCG2. It is evident from our accumulation and efflux studies that BMS599626 shows a concentration-dependent increase in accumulation caused by a decrease in efflux of [^3^H]-mitoxantrone, consistent with BMS-599626 blocking the transporter. There are various mechanisms by which inhibitors of ABC transporters exhibit their inhibitory effects [38]. To rule out the possibility of a decrease in the protein expression of ABCG2, we carried out immunoblotting, the results of which show that upon treatment with BMS-599626, the expression of ABCG2 over time (72 h) remained unchanged. Furthermore, there was no change in the localization of ABCG2 transporter from the cell membrane to the cytoplasm, suggesting that BMS-599626 does not show its reversal effect by decreasing the expression of ABCG2 protein or altering its cellular localization, but by inhibiting its efflux function.

ABC transporters function by utilizing the energy obtained by ATP hydrolysis [2,3,12]. The presence of a substrate or an inhibitor can alter the activity of this enzyme. Previous studies have shown that inhibitors of ABC transporters can either inhibit or promote ATPase activity [8,11,18]. BMS-599626 at a concentration of 3 µM produced a 7.6-fold inhibition of ATPase activity, indicating an interaction of BMS-599626 with the drug-binding site of ABCG2. The concentration for the inhibition of ATPase is 10 times higher compared to the chemosensitizing effect of BMS-599626, due to the difference in the models for both the experiments. While the chemosensitizing effect of BMS-599626 was determined in drug resistant cell lines, the ATPase assay was performed using membrane vesicles prepared from ABCG2 expressing High-five insect cells. Lastly, our in-silico study indicates that hydrophobic interactions lead to the stability of BMS-599626 at the drug binding site at the transmembrane domain of ABCG2. In addition, the low IFD Glide gscore corresponds to stronger binding affinity. In fact, BMS-599626 presented an IFD Glide gscore of −12.886 kcal/mol, which is better than that of other previously reported reversal compounds [10,11,18]. Thus, in aggregate, our results suggest that BMS-599626 potently inhibits the efflux function of ABCG2.

## 4. Materials and Methods 

### 4.1. Reagents

BMS-599626 was provided by Chemie-Tek (Indianapolis, IN, USA). Cell culture reagents: Dulbecco’s modified Eagle’s medium (DMEM), fetal bovine serum (FBS), penicillin/streptomycin and trypsin were acquired from Hyclone (GE Healthcare Life Science, Pittsburgh, PA, USA). Monoclonal antibodies against ABCG2 and GAPDH were obtained from Cell Signaling Technology (Danvers, MA, USA). Alexa Fluor 488 conjugated goat anti-rabbit IgG secondary antibody was purchased from Thermo Fisher Scientific Inc. (Rockford, IL, USA). Cisplatin, mitoxantrone and topotecan and positive control inhibitor of ABCG2 Ko143 were procured from Sigma-Aldrich (St. Louis, MO, USA). [^3^H]-mitoxantrone (2.5 Ci/mmol) was a product of Moravek Biochemicals, Inc (Brea, CA, USA).

### 4.2. Cell Lines and Cell Culture

The cancer cell line NCI-H460, and its mitoxantrone-selected drug-resistant sublines NCI-H460/MX20 and HEK293 cells transfected with either pcDNA3.1 empty vector (HEK293/pcDNA3.1) or wild-type ABCG2 (HEK/ABCG2-R482)-containing vector or its mutant ABCG2 (HEK/ABCG2-R482G and HEK/ABCG2-R482T) were used for current study of ABCG2 [16]. ABCB1 reversal study was carried out on the colorectal carcinoma cell line SW620 and its doxorubicin-resistant SW620/AD300 cell line, whereas the human epidermoid carcinoma cell subline KB-3-1 and its high ABCC1-expressing KB-CV60 cell subline were used for the ABCC1 reversal study, and HEK293/pcDNA3.1 and ABCC10-overexpressing HEK293/ABCC10 cells were used for the ABCC10 reversal study [18,39,40]. The HEK293 transfected cells were maintained in media containing G418 (2 mg/mL). All the other cell lines were cultured in DMEM containing FBS (10%) and penicillin-streptomycin (1%). The cell lines were incubated at 37 °C and 5% CO_2_.

### 4.3. Cytotoxicity Assay

The MTT assay was carried out to determine the cytotoxicity of BMS-599626 in parental and ABCB1-, ABCG2-, ABCC1- and ABCC10-overexpressing sublines. Cells were trypsinized, resuspended and 5 × 10^3^ cells per well were seeded in a 96-well plate. After 24 h, the cells were incubated with or without drugs for 72 h preceded by a 2 h treatment with either BMS-599626 or Ko143. MTT (3 mg/mL) was added to the cells and the cells were further incubated for another 4 h. Subsequently, the supernatant was removed and dimethyl sulfoxide (DMSO) was added to dissolve the formazan crystals. The absorbance was read in an Opsys microplate reader (Dynex Technologies, Chantilly, VA) at an absorbance of 570 nm. The concentration at which 50% of cell growth was inhibited (IC_50_) was determined as illustrated previously [8,18]. The ratio of the IC_50_ of the resistant cells to the IC_50_ of sensitive cells yielded the resistant folds (Rf). The concentrations of BMS-599626 used for the reversal studies were 100 and 300 nM. Ko143 was used as positive control for inhibition of ABCG2. Cytotoxicity assays were conducted in triplicates and each assay was run at least three times.

### 4.4. [^3^H]-Mitoxantrone Accumulation Assay

The parental and ABCG2-overexpressing cells were incubated with 0.01 µM radiolabeled-mitoxantrone for 2 h preceded by incubation with or without BMS-599626 at 100 or 300 nM or Ko143 at 300 nM. This was followed by washing the cells with PBS and lysing them with lysis buffer. The lysed cells were added to 5 mL scintillation fluid in scintillation vials. Radioactivity was detected using a Packard TRI-CARB 1900CA liquid scintillation analyzer.

### 4.5. [^3^H]-Mitoxantrone Efflux Assay

The reduction in efflux of radiolabeled mitoxantrone due to the inhibition of ABCG2 was determined by treating the cells with 0.01 μM [^3^H]-mitoxantrone for 2 h. The cells were then washed twice with cold PBS followed by incubation in DMEM with or without BMS-599626 at 100 or 300 nM or Ko143 at 300 nM. Subsequently, fractions of cell suspensions were collected at various time periods (0, 30, 60, and 120 min). The cells were then lysed with lysis buffer and analyzed as listed in Section 4.4.

### 4.6. Immunoblotting

Western blotting was performed to determine the change in the expression of ABCG2 upon treatment with the inhibitor. The cells were lysed following treatment with or without BMS-599626 at 100 or 300 nM and Ko143 at 300 nM at 0, 24, 48 and 72 h. After determining the protein content, equal 60 µg protein aliquots were loaded and resolved by SDS-PAGE and transferred to a PVDF membrane. The membrane was left for overnight incubation at 4 °C with primary monoclonal antibodies against ABCG2 (1:500 dilution) and GAPDH (1:1000). The membrane was then incubated with horseradish peroxide (HRP)-conjugated secondary antibody (1:1000) at room temperature for 2 h. Bands were detected using an ECL detection system, followed by analysis using ImageJ software (NIH, Bethesda, MD, USA).

### 4.7. Immunofluorescence Assay

The parental and ABCG2-overexpressing cells were seeded at a density of 1×10^4^ cells per well in 24-well plates and the effect of BMS-599626 on the cell surface expression of ABCG2 was assessed using the immunofluorescence assay as illustrated previously [15,18]. 

### 4.8. ATPase Assay

The membrane vesicles of insect cells containing ABCG2 were treated with BMS-599626 (0–40 µM final concentration) and the ATPase activity was measured as illustrated previously [11,41]. 

### 4.9. Molecular Docking Analysis

All molecular docking simulations were carried out in Maestro v11.1 (Schrödinger, LLC, New York, NY, USA) on a 6-core Xeon processor with a MacOS. The ligand and protein were prepared as described previously [36]. The cryo-EM structure of human ABCG2 (PDB ID: 6ETI) was used in this study, and the molecular modeling grid was generated at the drug interacting site by choosing primary residues (including Ala397, Val401, Leu405, Leu539, Ile547) [14]. The binding poses of BMS-599626 in human ABCG2 from Glide docking (Glide v 7.4, extra precision) were ranked by the Glide E model value to pick a best docked pose, which was then subject to induced-fit docking (IFD) to get a more accurate binding simulation. The best scored docking pose simulated by IFD was analyzed. The molecular modeling hits were measured and represented as kcal/mol.

### 4.10. Statistical Analysis

The experimental results are presented as the mean of three or more individual experiments. Deviations from the mean are presented as mean ± SD. ANOVA was used to determine a statistical significance of *p* < 0.05.

## 5. Conclusions

BMS-599626, a pan-HER kinase inhibitor, is currently being investigated in patients with advanced solid malignancies. The findings of our study, for the first time, show that BMS-599626, at concentrations as low as 300 nM, antagonizes ABCG2-mediated MDR. Our results demonstrate that BMS-599626 increases the sensitivity of ABCG2-overexpressing cells to the substrates of ABCG2 and enhances the drug accumulation of ABCG2 substrates. BMS-599626 was found to inhibit the drug efflux activity of ABCG2 without altering the expression of ABCG2 protein or its cellular localization. We hereby propose that BMS-599626 be considered for use in combination with substrates of ABCG2 to improve cancer therapy.

## Figures and Tables

**Table 1:** The effect of BMS-599626 on reversal of ABCB1, ABCC1, and ABCC10-mediated MDR.

**Treatment**	**IC_50_ ± SD ^a^ (μM) (FR ^b^)**	
	**SW620**	**SW620/AD300**
Doxorubicin	0.09 ± 0.006 (1)	20.58 ± 2.11 (228.67)
+100 nM BMS-599626	0.09 ± 0.009 (1)	19.85 ± 1.08 (220.55)
+300 nM BMS-599626	0.09 ± 0.008 (1)	17.67 ± 2.01 (196.33)
+300 nM Verapamil	0.09 ± 0.009 (1)	5.95 ± 0.06 (66.11) *
**Treatment**	**IC_50_ ± SD ^a^ (μM) (FR ^b^)**	
	**KB-3-1**	**KB-CV60**
Vincristine	0.09 ± 0.009 (1)	41.99 ± 4.23 (446.55)
+100 nM BMS-599626	0.09 ± 0.004 (1)	31.57 ± 3.27 (350.77)
+300 nM BMS-599626	0.08 ± 0.006 (0.89)	29.82 ± 3.01 (331.33)
+25 μM MK571	0.09 ± 0.008 (1)	3.09 ± 0.04 (34.33) *
**Treatment**	**IC_50_ ± SD ^a^ (μM) (FR ^b^)**	
	**HEK293/pcDNA3.1**	**HEK293/ABCC10**
Vincristine	0.08 ± 0.009 (1)	1.09 ± 0.02 (13.62)
+100 nM BMS-599626	0.09 ± 0.10 (1.1)	0.61 ± 0.005 (7.62)
+300 nM BMS-599626	0.07 ± 0.008 (0.87)	0.50 ± 0.007 (6.25)
+300 nM Cepharathine	0.07 ± 0.006 (0.87)	0.23 ± 0.003 (2.87) *

^a^ IC_50_ values are represented as mean ± SD of at least three independent experiments performed in triplicates. ^b^ FR: Resistance fold is the ratio of the IC_50_ values of substrates in the presence or absence of inhibitor to the IC_50_ of parental cells without inhibitor. (*) specifies *p* < 0.05 compared to control group.

## Supplementary Materials

The following are available online at https://www.mdpi.com/2072-6694/12/9/2502/s1. Figure S1: Whole uncut blots for Figure 6; Table S1: Intensity ratio of the bands for NCI-H460 and NCI-H460/MX20 cells treated with BMS-599626; Table S2: Intensity ratio of the bands for HEK293/pcDNA3.1 and HEK/ABCG2-R482G cells treated with BMS-599626.
