# Supplementary material for: BMS-599626, a Highly Selective Pan-HER Kinase Inhibitor, Antagonizes ABCG2-Mediated Drug Resistance
Source: Cancers (Basel). 2020 Sep 3;12(9):2502. doi: 10.3390/cancers12092502 (PMC7565406; doi:10.3390/cancers12092502)
Supplement: Supplementary file 1 [file cancers-12-02502-s001.pdf]

Supplemental Materials

BMS-599626, a Highly Selective Pan-HER Kinase Inhibitor, Antagonizes ABCG2-Mediated Drug Resistance

Yunali V. Ashar, Jingchun Zhou, Pranav Gupta, Qiu-Xu Teng, Zi-Ning Lei, Sandra E. Reznik, Sabrina Lusvarghi, John Wurpel, Suresh V. Ambudkar and Zhe-Sheng Chen

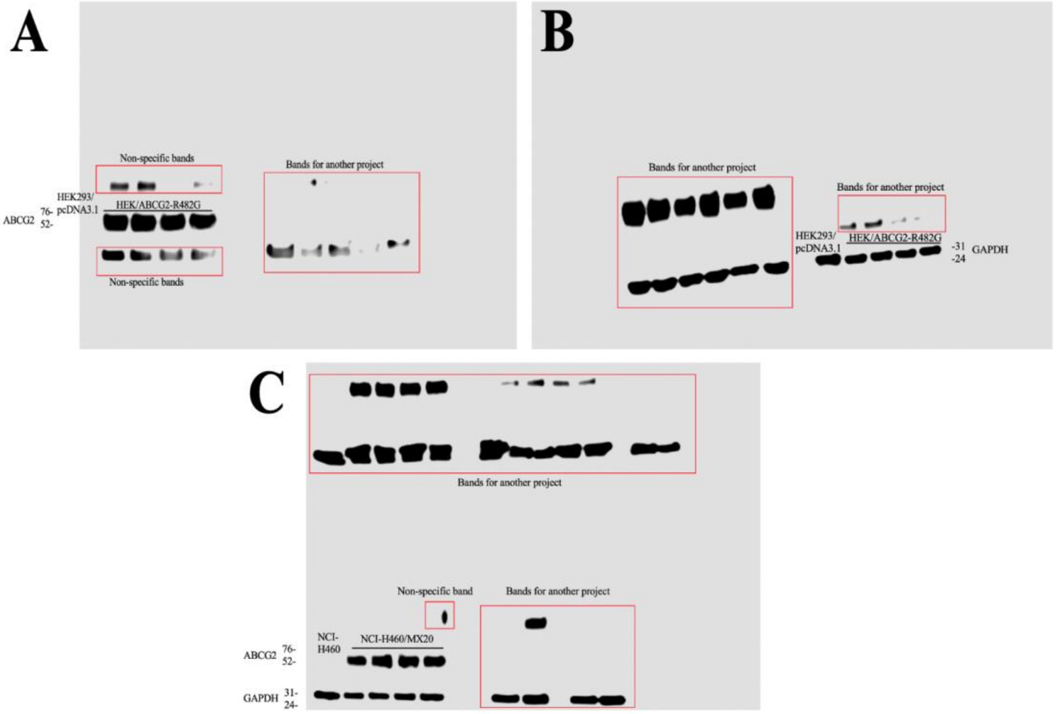

Figure S1. Whole uncut blots for Figure 6.

Table S1. Intensity ratio of the bands for NCI-H460 and NCI-H460/MX20 cells treated with BMS-599626.

Intensity/ Area	NCI-H460	NCI-H460/MX20-0 h	NCI-H460/MX20-24 h	NCI-H460/MX20-48 h	NCI-H460/MX20-72 h
ABCG2	4321.92	33962.18	33740.68	32278.35	29962.32
GAPDH	29281.37	21766.28	21791.1	18642.57	20249.15
Intensity Ratio	0.1476	1.560312	1.54837	1.731432	1.479682

Table S2. Intensity ratio of the bands for HEK293/pcDNA3.1 and HEK/ABCG2-R482G cells treated with BMS-599626.

Intensity/Area	HEK293/pcDNA3.1	HEK/ABCG2-R482G -0 h	HEK/ABCG2-R482G -24 h	HEK/ABCG2-R482G -48 h	HEK/ABCG2-R482G -72 h
ABCG2	2519.87	19796.33	22259.64	21857.88	22134.88
GAPDH	32416.9	19868.98	20929.57	20606.74	21908.3
Intensity Ratio	0.077733	0.996343	1.06355	1.060715	1.010342
